# Gait Asymmetry in Arm Swing and Foot Progression Angle of Knee Osteoarthritis

**DOI:** 10.3390/jcm15145360

**Published:** 2026-07-09

**Authors:** Ji-Yeon Yoon, Sang Won Moon

**Affiliations:** 1 Department of Physical Therapy, Inje University Haeundae Paik Hospital, Busan 48108, Republic of Korea; yoonzzi@paik.ac.kr; 2 Department of Orthopedic Surgery, Inje University Haeundae Paik Hospital, 875 Haeun-Daero, Haeundae-gu, Busan 48108, Republic of Korea

**Keywords:** knee osteoarthritis, arm swing, gait asymmetry

## Abstract

**Background**: Knee pain, proprioceptive deficits, and muscle weakness brought about by articular degeneration in knee osteoarthritis (OA) can cause postural instability and gait asymmetry. Arm swing plays a major role in balance control during human walking. However, few studies have examined the arm movement and gait stability in knee OA. Therefore, the purpose of this study is to investigate the gait asymmetry in upper and lower limb movement and to understand the overall movement pattern during walking in people with knee osteoarthritis. **Methods**: Thirty-five people with knee OA and twenty-four age-matched controls were enrolled in this study. The arm swing amplitude, gait biomechanics, and spatiotemporal parameters during walking were measured using a Vicon motion capture system incorporating two AMTI force plates. The differences between the knee OA and control groups were analyzed using independent *t*-tests. **Results**: The knee OA patients walked with a slower, smaller step and lower arm swing amplitude. The asymmetries of step time, foot progression angle, and total arm swing amplitude were significantly greater in the knee OA group than in the controls (*p* < 0.05). There was no significant difference in the mean value of step width and the foot progression angle and the asymmetry of step length and the knee adduction moment between the groups. **Conclusions**: This study demonstrates that the pathological changes in the lower limbs in knee OA are intrinsically reflected in the upper limbs through diagonal coordination. Asymmetry of arm swing and foot progression angle may be key for understanding OA-related instability.

## 1. Introduction

The human body is functionally divided into a locomotor and passenger unit during walking. For normal gait, the two lower limbs and pelvis act as a locomotor unit and must have stance stability, shock absorption, energy conservation, and progression. In particular, the knee joint plays a major role in shock absorption during weight acceptance [1]. Aging and pathological lesions such as knee arthritis (OA) could affect dynamic stability and change gait patterns by failing to perform key functions properly [2,3]. Knee OA is a widespread progressive joint disease among elderly people that causes gait instability due to pain, deformity, stiffness, and muscle weakness [4,5,6]. Patients with knee OA adopt compensatory strategies such as altered foot progression angle and gait asymmetry as well as slower walking speed and shorter step length with decreased joint motions. In addition, they also change trunk and upper limb movement, such as by flexing the trunk and lateral tilting [7,8,9,10].

As a part of the passenger unit, the trunk and upper limbs are responsible for balance control and gait efficiency [11]. In particular, arm swing provides a purposeful counterforce to minimize energy consumption by reducing ground reaction force during weight acceptance and can help restore balance after a perturbation to optimize stability [12]. Reciprocal arm swing is rhythmical movement via neuronal control of the upper and lower limbs during human walking [1]. Arm swing amplitude decreases with older age, and limited arm swing increases energy consumption and risk of falling with unstable walking [12,13]. Changes in arm positions and movement may affect interlimb coordination.

Previous research found that gait asymmetry is one symptom of knee OA gait that reduces gait stability. Iijima et al. [14] suggested that medio-lateral trunk movement asymmetry can be used to detect asymmetric gait, and Wang et al. [9] concluded asymmetric gluteus medius muscle force may be an indicator in knee OA patients. Creaby et al. [15] found that gait asymmetry is more symptom-dependent rather than structural lesion status, and unilateral pain increases asymmetry. Studies on the gait efficiency and asymmetry of upper and lower limbs during walking in knee OA are still lacking. Until now, most walking studies in knee OA patients have focused on the lower extremities. Although arm movement and the trunk play an important role in walking, few studies have been conducted. In addition, since it is limited to partial movement studies, further investigation is needed to understand the overall movement pattern during walking in patients with knee OA and to provide information on postoperative gait rehabilitation by analyzing overall movement patterns such as in lower extremities and interbody and arm movements. Therefore, this study aimed to investigate the differences in gait asymmetry in arm swing as well as lower limb movement. We hypothesized that arm swing amplitude would be smaller with a slower walking speed in knee OA and that the asymmetry in arm swing and KAM would be noticeable.

## 2. Methods

### 2.1. Subjects

This cross-sectional comparative study enrolled 35 patients with moderate to severe medial knee OA who were waiting to undergo total knee arthroplasty (TKA) surgery. The sample size was calculated using G-Power software (version 3.1.9.7; Franz Faul, University of Kiel, Germany). The effect size determined from a pilot study with 7 people in each group was 2.6. The calculated sample size was 12 or 6 for each group (α = 0.05, effect size: 2.6, power of 95%). The inclusion criteria were as follows: (1) aged 60 years or older, (2) diagnosed with knee OA in the medial compartment, classified as Kellgren–Lawrence grade 3 (moderate) or 4 (severe). The exclusion criteria were as follows: concurrent shoulder and back pain or any neurological disorders that could affect stable gait, and more osteophytes in the lateral compartment. Twenty-four age-matched healthy elderly people were recruited as control group. The inclusion criteria were as follows: aged over 60 years, no clinical diagnosis of knee OA, no neurological disorders or musculoskeletal disease, and no history of upper and lower limb surgery. This study was conducted at Inje University Haeundae Paik Hospital, Busan, Republic of Korea, between November 2021 and August 2023. Participants were enrolled consecutively if they met the inclusion criteria and provided written informed consent; no participants were excluded after screening. IRB approval was obtained prior to data collection. The reporting of this study conforms to the STROBE guidelines [16] (see Supplementary Materials).

### 2.2. Measurement Protocol

Gait parameters including kinetic and kinematic data were captured and analyzed using a VICON motion capture system (VICON Motion Systems Ltd., Oxford, UK). Prior to the test, 20 retro-reflective markers (diameter of 14 mm) were placed on each subject. The locations of the markers were based on the modified Plug-in-Gait model marker. Sixteen markers were set for locomotion unit motion (bilateral anterior and posterior superior iliac spines, lateral thigh, femoral epicondyle, tibia malleolus, second metatarsal head, and posterior calcaneus), and four markers were bilaterally attached on the acromion processes and lateral epicondyle of the humerus for the arm swing motion. All markers were attached by the same examiner. First, a static standing posture was captured, and then, each subject was asked to walk at a natural speed along an 8 m walkway with two ground-embedded force plates (AMTI, Advanced Mechanical Technology Inc., Watertown, MA, USA) in the middle.

The marker data were synchronized with the kinematic data via Nexus software (version 1.7, VICON Motion Systems Ltd., Oxford, UK) and filtered at 6 Hz using a zero-lag, bidirectional second-order Butterworth filter. Spatiotemporal parameters and kinematic and kinetic data were calculated for each subject according to the anthropometric characteristic. Spatiotemporal parameters were included: walking speed, step length, step time, and step width. Step length was normalized to the height. Foot progression angle was calculated as the mean value during stance phase. The kinetic data, including the knee adduction moment (KAM), were recorded using two force plates with a sampling rate of 1000 Hz and processed using a low-pass filter with a cut-off frequency of 50 Hz. Only data on which each foot was precisely stepped on the force plate were selected and analyzed. Peak external KAM was identified during stance phase. The acquired gait data were analyzed using Polygon software (version 3.1, VICON Motion Systems Ltd., Oxford, UK), and the gait data were time normalized to the gait cycle. The KAM was normalized for body weight and height (%BW·Ht). The arm swing motions were calculated using the angle between the arm and virtual vertical axis and divided into the anteversion and retroversion of the shoulder joint. Total arm swing amplitude was defined for the maximum value as peak shoulder flexion motion and for the minimum value as peak shoulder extension motion and calculated as the range (maximum–minimum values) during one gait cycle, and the values of four gait cycles were averaged. The symmetry angle (SA) was used to evaluate the asymmetry of the gait data. The equation proposed by Zifchock et al. [17] is the following:

$$\mathrm{SA}=\left|\frac{45{}^{\circ}-arctan\left(\frac{X_{L\;\mathrm{or}\;L}}{X_{M\;\mathrm{or}\;R}}\right)}{90{}^{\circ}}\right|\;\times \;100\;(\%)$$

where X_M_ and X_L_ are the angles of the more (M) and less (L) affected side in knee OA, and X_R_ and X_L_ are the angles of the right (R) and left (L) side in controls, respectively. Higher SA values indicate more asymmetry.

### 2.3. Statistical Analysis

SPSS software (version 23.0, IBM Corp., Armonk, NY, USA) was used for statistical analyses. Levene’s test was used to assess for equality of variance, and the Shapiro–Wilk test was used to analyze the normality of the data distribution. Differences in the general participant characteristics, gait parameters, and asymmetry of gait data between two groups were compared using independent *t*-tests. After controlling height, weight, and BMI, analysis of covariance (ANCOVA) was performed to compare group differences in arm swing, gait parameters, and asymmetric data. R software (version 4.6.0; https://www.r-project.org/) was used for multiple comparisons. Benjamini–Hochberg’s False Discovery Rate for multiple comparisons was applied to evaluate which group received the more effective intervention. The statistical significance level was accepted at *p* < 0.05 and the confidence interval was kept at 95%. Cohen’s d was used to evaluate the effect size between two groups. All enrolled participants completed the protocol; no missing data were present.

## 3. Results

### 3.1. Subjects’ Characteristics

The baseline demographics of the two groups are summarized in Table 1. The age and gender ratio were not significantly different between the groups; however, height, weight, BMI, and knee varus alignment showed significant differences between groups (*p* < 0.05). The mean (SD) in Table 2 and Table 3 is the mean ± standard deviation before the covariates (height, weight, BMI) were input, and the adjusted mean difference between groups and statistical significance were corrected by inputting the covariates. And B-H stands for Benjamini–Hochberg False Discovery Rate (FDR), which determines statistical significance based on this value.

### 3.2. Gait and Arm Swing Data

Significant decreases in walking speed and step length were observed in the knee OA group compared to the control group (*p* < 0.05). Conversely, step time significantly increased in the knee OA group compared to the control group (*p* < 0.05) (Table 2). In the knee OA group, the peak external KAM was higher on the more affected side than on the less affected side. Furthermore, the KAM of the more affected side in the knee OA group was significantly higher than that of the right side in the control group (*p* < 0.05) and the foot progression angle of less affected side in the knee OA was significantly lower than that of the left side in the control group (*p* < 0.05). There were no significant differences in KAM between the less affected side in the knee OA group and the left side in the control group and in foot progression angle of the more affected side in the knee OA group and the right side in the control group (Table 2). The total arm swing amplitudes of both sides in the knee OA group were significantly smaller than those in the controls (*p* < 0.05) (Table 2).

### 3.3. Asymmetry in Gait and Arm Swing

The left-right asymmetries of step time and foot progression angle were significantly greater in the knee OA than in the control group (*p* < 0.05) (Table 3). The asymmetry of arm swing tended to increase significantly in knee OA, although the *p* value is 0.059 when correction measures are applied, it is close to the significance and the *p* value is 0.034 when ANCOVA is applied, so it can be said that there is a significant difference. In contrast, the asymmetry of the step length and KAM did not significantly differ between the groups.

## 4. Discussion

The primary objective of this study was to elucidate the alterations in gait biomechanics and kinematic asymmetries, specifically focusing on the between arm swing and lower limbs in patients with knee OA. Our findings reveal that knee OA patients employ a distinct “safety-first” gait strategy, characterized by reduced walking velocity and shortened step length. Notably, this pathological gait was accompanied by a significant reduction in arm swing amplitude. Furthermore, while the mean values of the foot progression angle (FPA) remained comparable to controls, the knee OA group exhibited markedly higher asymmetries in step parameters, arm swing, and FPA.

The observed reduction in arm swing amplitude is closely linked to the decreased step length and gait speed inherent in knee OA [18]. Since the diagonal coupling between the upper and lower extremities is neurologically regulated by central pattern generators (CPGs) to minimize total body angular momentum, any disruption in lower limb kinematics inevitably alters arm swing dynamics [19,20]. Consequently, our results suggest that impaired arm swing in knee OA is not merely a byproduct of pain but a failure to optimize COM stability through effective trunk-limb coordination.

The KAM is a primary indicator of medial and lateral loading distribution and higher KAM is the strongest biomechanical risk factor for medial knee OA [21,22]. The KAM of more affected side in knee OA is significantly greater than it of controls, meanwhile it of less affected side did not differ. Knee OA patients walk slowly with a antalgic or limping gait which is pain-related behavior [23]. The subjects of this study also showed a limping gait with shorter step length, which can be confirmed in the asymmetry of step time. Their arm swing amplitude also decreased and the asymmetry between both arms increased compared to the controls. Although handness are not associated with asymmetry of arm swing, footedness in healthy elderly could affect asymmetry of arm swing during walking [24,25]. In this study, knee OA patients showed further reduced arm swing amplitude of contralateral side, indicating that their arm-leg coordination was well maintained. We consider that knee OA patients may walk in their affected side by transferring load on their contralateral side to minimize pain, therefore their footedness may cause the asymmetry of arm swing. The absence of a significant difference from the control in asymmetry between the two KAMs also seems to be associated with shifting the weight to the opposite leg. The KAM is determined by vertical ground reaction force (v-GRF) and moment arm [11,26], the v-GRF of more effected side in knee OA could be lower due to the pain and weakness and the moment arm could be greater, on the other hand; the contralateral side would have the opposite result. Therefore, it is thought that the asymmetry of each individual’s left and right KAM was not significantly different from that of the control group. Wang et al. [9] reported that the difference in peak KAM between left and right appeared in the mild stage of Knee OA.

The asymmetry angle is more affected by directionality than by the value. All knee OA patients of this study had knee varus angle (positive value) during standing and walking. Meanwhile, 5 people in the control group showed valgus angle (negative value) on one side. Therefore, control group showed the greater asymmetry of the knee varus angle during standing and walking. Since the deviation of the knee angle when walking was greater than in the standing posture, there was a significant difference between the two groups in the asymmetry of the knee angle when walking. This deviation was also shown in FPA, but the result was the opposite. In knee OA patients, 12 patients showed the opposite left-right angle pattern, meanwhile only one was in the control group. Therefore, there was a significant difference in asymmetry of FPA. Another strategy for patients with knee OA to reduce the medial knee joint load is to change the direction of the foot [7]. In a situation where the moment arm increases as the knee varus angle increases, the moment arm is moved outward by turning the toe-out to move the center of pressure outward to reduce the moment arm in the knee [10,27,28]. Chen et al. [29] said that in knee OA patients had more severe asymmetry in foot posture. Knee OA patients had a large deviation such as toe-in or toe-out, so it is considered that there was no significant difference between groups.

This study has some limitations. First, despite matching for age and sex, differences in BMI may have influenced the results as obesity is a critical factor in knee OA. Hence Height, weight, and BMI were adjusted in statistical analysis and results were derived. Second, the sample size was relatively small and uneven because the subjects were patients scheduled for surgery, which may limit the generalizability of the findings to mild OA cases. Third, although pain is associated with gait asymmetry, pain severity or function score could not be measured. Fourth, although arm swing is closely related to energy consumption, we did not measure direct metabolic consumption. Lastly, while FPA was measured, a comprehensive evaluation of foot posture was not conducted. Future studies should be conducted on more Knee OA patients well classified into pain and functional evaluation, and the relationship between body and pelvis coordination and energy recovery should also be studied.

## 5. Conclusions

This study demonstrates that the pathological changes in the lower limbs in knee OA are intrinsically reflected in the upper limbs through diagonal coordination. The “safety-first” gait adopted by these patients is characterized by slower, smaller steps and arm swing. People with knee OA may change the arm swing amplitude and foot progression angle to compensate the gait instability. Asymmetry of arm swing and foot progression angle may be key for understanding OA-related instability. Our findings suggest that clinical interventions should extend beyond pain management to include gait training that restores symmetric limb coordination patterns, which is essential for optimal functional recovery after knee surgery.

## Figures and Tables

**Table 1:** Subjects’ characteristics.

	Knee OA (*n* = 35)	Controls (*n* = 24)	
	Mean (SD)	Mean (SD)	*p*
Age (years)	70.7 (6.0)	71.7 (6.5)	0.565
Height (m)	1.57 (0.08)	1.61 (0.08)	**0.040**
Weight (kg)	67.2 (10.8)	60.2 (9.3)	**0.013**
BMI (kg/m^2^)	27.3 (4.0)	23.3 (3.6)	**<0.000**
Knee varus alignment (MAS/R) (°)	9.94 (4.06)	0.24 (1.51)	**<0.000**
Knee varus alignment (LAS/L) (°)	6.18 (5.02)	0.25 (1.60)	**<0.000**
Female:male	26:9	17:7	

MAS, more affected side; LAS, less affected side; R, right; L, left; BMI, body mass index; SD, standard deviation. Bold type represents a statistically significant result.

**Table 2:** Gait and arm swing data.

	Knee OA (*n* = 35)	Controls (*n* = 24)	Adjusted Mean Difference Mean	Adjusted *p*-Value/ B-H	Effect Size (95% CI)
	Mean (SD)	Mean (SD)			
**Spatiotemporal parameters**					
Walking speed (m/s)	0.71 (0.28)	1.01 (0.11)	−0.243	**<0.001/** **0.001**	−1.173 (−1.826–−0.521)
Step time (s)	0.66 (0.12)	0.55 (0.04)	0.113	**<0.001/** **0.001**	1.145 (0.494–1.796)
Step length/height ratio	0.28 (0.07)	0.35 (0.03)	−0.053	**0.001/** **0.002**	−0.892 (−1.528–−0.256)
Step width (m)	0.16 (0.05)	0.14 (0.04)	0.013	0.345/ 0.401	0.291 (−0.324–0.906)
**Kinetic and kinematics**					
Knee adduction angle (MAS/R) (°)	18.84 (8.21)	−2.68 (4.33)	23.836	**<0.001** **<0.001**	3.532 (2.772–4.291)
Knee adduction angle (LAS/L) (°)	21.06 (7.50)	−2.79 (3.85)	26.800	**<0.001/** **<0.001**	5.037 (4.150–5.925)
Knee adduction moment (MAS/R) (% Ht*BW)	3.57 (1.39)	2.70 (0.81)	1.018	**0.004/** **0.007**	0.855 (0.221–1.490)
Knee adduction moment (LAS/L) (% Ht*BW)	2.61 (1.21)	2.34 (0.98)	0.303	0.376/ 0.401	0.273 (−0.342–0.887)
Foot progression angle (MAS/R) (°)	7.99 (7.59)	7.13 (4.34)	−0.367	0.855/ 0.852	−0.056 (−0.669–0.556)
Foot progression angle (LAS/L) (°)	7.29 (6.76)	9.78 (4.69)	−4.026	**0.030/** **0.048**	−0.682 (−1.309–−0.056)
**Arm swing**					
Total arm swing amplitude (MAS) (°)	14.01 (8.47)	26.07 (9.54)	−9.069	**0.001/** **0.002**	−1.067 (−1.713–−0.421)
Total arm swing amplitude (LAS) (°)	12.31 (8.70)	25.08 (9.14)	−12.168	**<0.001/** **<0.001**	−1.348 (−2.014–−0.683)

MAS, more affected side; LAS, less affected side; R, right; L, left; BW, body weight; Ht, height; SD, standard deviation. Bold type represents a statistically significant result.

**Table 3:** Asymmetric data.

	Knee OA (*n* = 35)	Controls (*n* = 24)	Adjusted Mean Difference Mean	Adjusted *p*-Value/ B-H	Effect Size (95% CI)
	Mean (SD)	Mean (SD)			
Knee varus alignment	24.29 (25.15)	40.74 (43.98)	−5.586	0.571/ 0.571	−0.166 (−0.780–0.447)
Step length	2.47 (2.88)	1.34 (1.14)	0.949	0.104/ 0.145	0.400 (−0.217–1.017)
Step time	2.58 (2.07)	1.02 (1.15)	1.397	**0.011/** **0.026**	0.808 (0.176–1.440)
Arm swing	13.00 (9.91)	8.30 (5.69)	5.747	**0.034/** 0.059	0.666 (0.040–1.292)
Knee adduction angle	26.60 (8.34)	53.10 (4.27)	−29.778	**<0.001/** **<0.001**	−5.037 (−6.186–−3.889)
Knee adduction moment	12.37 (11.33)	10.05 (8.40)	2.960	0.353/ 0.412	0.286 (−0.329–0.901)
Foot progression angle	47.07 (41.93)	19.00 (9.42)	25.388	**0.002/** **0.007**	0.767 (0.137–1.397)

SD, standard deviation; B-H, Benjamini–Hochberg; CI, confidence interval. Bold type represents a statistically significant result.

## Data Availability

The data presented in this study are available on request from the corresponding author. The data are not publicly available due to privacy and ethical restrictions regarding participant confidentiality.

## Supplementary Materials

The following supporting information can be downloaded at: https://www.mdpi.com/article/10.3390/jcm15145360/s1 [16].
