# Supplementary material for: Gait Asymmetry in Arm Swing and Foot Progression Angle of Knee Osteoarthritis
Source: J Clin Med. 2026 Jul 9;15(14):5360. doi: 10.3390/jcm15145360 (PMC13410256; doi:10.3390/jcm15145360)
Supplement: Supplementary file 1 [file jcm-15-05360-s001.zip › jcm-4351488-supplementary.pdf]

STROBE Statement — Checklist of items that should be included in reports of cross-sectional studies

Manuscript: Gait asymmetry in arm swing and foot progression angle of knee osteoarthritis

Journal of Clinical Medicine (MDPI) | Manuscript ID: jcm-4351488

Status: YES = Item fully reported | N/A = Not applicable to this study design

Section / Item	No.	Recommendation	Reported in manuscript	Status
**TITLE AND ABSTRACT**				
**Title and abstract**	1(a)	Indicate the study's design with a commonly used term in the title or the abstract	Abstract (Methods): "Thirty-five people with knee OA and 24 age-matched controls were enrolled." Study design identified as cross-sectional comparative study in Methods section.	YES
	1(b)	Provide an informative and balanced summary of what was done and what was found	Abstract: Background, Methods, Results, and Conclusions all reported with key findings (arm swing reduction, gait asymmetry, FPA asymmetry).	YES
**INTRODUCTION**				
**Background / rationale**	2	Explain the scientific background and rationale for the investigation being reported	Introduction: Pathophysiology of knee OA, role of arm swing in gait stability, and gap in literature on upper-lower limb asymmetry in OA gait described.	YES
**Objectives**	3	State specific objectives, including any prespecified hypotheses	Introduction (final paragraph): "This study aimed to investigate the differences of gait asymmetry in arm swing as well as lower limbs' movement." Hypothesis stated: arm swing amplitude would be smaller and asymmetry in arm swing and KAM would be noticeable.	YES
**METHODS**				
**Study design**	4	Present key elements of study design early in the paper	Methods, Section 1 (Subjects): "This cross-sectional comparative study enrolled 35 patients..."	YES
**Setting**	5	Describe the setting, locations, and relevant dates, including periods of recruitment, exposure, follow-up, and data collection	Methods, Section 1 (Subjects): "This study was conducted at Inje University Haeundae Paik Hospital, Busan, Republic of Korea, between March 2021 and August 2023."	YES
**Participants**	6(a)	Give the eligibility criteria, and the sources and methods of selection of participants	Methods, Section 1: Inclusion criteria (age ≥60 yrs, KL grade 3–4 medial knee OA) and exclusion criteria (shoulder/back pain, neurological disorders, lateral osteophytes) stated for both OA (n=35) and control groups (n=24).	YES
	6(b)	For matched studies — give matching criteria and number of exposed and unexposed	Methods, Section 1: Age-matched controls recruited. Gender ratio confirmed non-significant between groups (Table 1).	YES
**Variables**	7	Clearly define all outcomes, exposures, predictors, potential confounders, and effect modifiers	Methods, Section 2: All outcome variables defined — arm swing amplitude, spatiotemporal parameters (walking speed, step length/time/width), KAM, foot progression angle, symmetry angle (SA). Equation for SA provided.	YES
**Data sources / measurement**	8	For each variable of interest, give sources of data and details of methods of assessment	Methods, Section 2: Vicon motion capture system, 20 retro-reflective markers (Plug-in-Gait model), AMTI force plates (1,000 Hz), Nexus software (v1.7), Polygon software (v3.1). Filtering parameters (6 Hz Butterworth, 50 Hz low-pass) specified.	YES
**Bias**	9	Describe any efforts to address potential sources of bias	Discussion (Limitations): BMI difference acknowledged; height, weight, BMI adjusted via ANCOVA. All markers attached by the same examiner to minimise measurement bias.	YES
**Study size**	10	Explain how the study size was arrived at	Methods, Section 1: Sample size calculated with G-Power v3.1.9.7; effect size 2.6 from pilot study (n=7/group), α=0.05, power=95%; required n=12 per group. Enrolled 35 OA and 24 controls.	YES
**Quantitative variables**	11	Explain how quantitative variables were handled in the analyses	Methods, Section 3: Continuous variables compared with independent t-tests; ANCOVA applied controlling for height, weight, BMI. Symmetry angle formula provided. Step length normalised to height.	YES
**Statistical**	12(a)	Describe all statistical	Methods, Section 3: SPSS v22.0; Levene's test (equality of variance); S	YES

Section / Item	No.	Recommendation	Reported in manuscript	Status
**methods**		methods, including those used to control for confounding	hapiro-Wilk test (normality); independent t-tests; ANCOVA (controlling height, weight, BMI); Benjamini-Hochberg FDR; Cohen's d. Significance level p<0.05, 95% CI.	
	**12(b)**	Describe any methods used to examine subgroups and interactions	Not applicable — no subgroup analyses performed.	N/A
	**12(c)**	Explain how missing data were addressed	Methods, Section 3: "All enrolled participants completed the protocol; no missing data were present."	YES
	**12(d)**	Cross-sectional study — describe analytical methods taking account of sampling strategy	Methods, Section 1: Consecutive enrolment described. ANCOVA applied to adjust for confounders (height, weight, BMI). Benjamini-Hochberg FDR applied for multiple comparisons.	YES
	**12(e)**	Describe any sensitivity analyses	Methods, Section 3: Benjamini-Hochberg FDR correction applied alongside ANCOVA as a sensitivity measure for multiple comparisons.	YES
**RESULTS**				
**Participants**	**13(a)**	Report numbers of individuals at each stage of study	Methods, Section 1 & Results, Section 3.1: 35 OA patients and 24 controls enrolled and analysed. Table 1 provides full baseline demographics.	YES
	**13(b)**	Give reasons for non-participation at each stage	Methods, Section 1: "Participants were enrolled consecutively if they met the inclusion criteria and provided written informed consent; no participants were excluded after screening."	YES
	**13(c)**	Consider use of a flow diagram	No participants were excluded after screening; all consecutive eligible patients who consented were included. A flow diagram was therefore not considered necessary.	YES
**Descriptive data**	**14(a)**	Give characteristics of study participants and information on exposures and potential confounders	Results, Section 3.1 & Table 1: Age, sex, height, weight, BMI, knee varus alignment reported for both groups with p-values.	YES
	**14(b)**	Indicate number of participants with missing data for each variable of interest	Methods, Section 3: "All enrolled participants completed the protocol; no missing data were present."	YES
**Outcome data**	**15**	Report numbers of outcome events or summary measures	Results, Sections 3.2–3.3 & Tables 2–3: Mean ± SD, adjusted mean difference, adjusted p-value/BH, and effect size (95% CI) reported for all gait, arm swing, and asymmetry parameters.	YES
**Main results**	**16(a)**	Give unadjusted estimates and confounder-adjusted estimates and their precision (95% CI)	Tables 2–3: Unadjusted means (Mean±SD) and ANCOVA-adjusted mean differences with 95% CI reported. Covariates (height, weight, BMI) specified in Methods and Results.	YES
	**16(b)**	Report category boundaries when continuous variables were categorised	Not applicable — no continuous variables were categorised.	N/A
	**16(c)**	If relevant, consider translating estimates of relative risk into absolute risk	Not applicable — this is not a risk estimation study.	N/A
**Other analyses**	**17**	Report other analyses done (subgroups, interactions, sensitivity analyses)	Results, Section 3.3: Both ANCOVA-adjusted p-values and Benjamini-Hochberg FDR values reported for all asymmetry measures. Arm swing asymmetry result discussed with both statistics (p=0.034 ANCOVA; BH =0.059).	YES
**DISCUSSION**				
**Key results**	**18**	Summarise key results with reference to study objectives	Discussion (first paragraph): "Our findings reveal that knee OA patients employ a distinct 'safety-first' gait strategy..." Key results (reduced arm swing, asymmetry in step time, FPA, arm swing) summarised.	YES
**Limitations**	**19**	Discuss limitations of the study, taking into account sources of potential bias or imprecision	Discussion (Limitations paragraph): Five limitations explicitly stated — (1) BMI differences, (2) small/uneven sample size, (3) absence of pain/function scores, (4) no metabolic measurement, (5) no comprehensive foot posture evaluation.	YES
**Interpretation**	**20**	Give a cautious overall	Discussion: Results interpreted in context of CPG theory and diagonal	YES

Section / Item	No.	Recommendation	Reported in manuscript	Status
		interpretation of results considering objectives, limitations, and other r elevant studies	coordination; compared with Wang et al. [9], Iijima et al. [14], Creaby et al. [15]. Limitations acknowledged throughout.	
**Generalisability**	**21**	Discuss the generalisa bility (external validity) of the study results	Discussion (Limitations): Findings noted to be limited to moderate-to-se vere OA patients awaiting TKA surgery, limiting generalisability to mild OA cases.	**YES**
**OTHER INFORMATION**				
**Funding**	**22**	Give the source of fund ing and the role of the f unders	Funding section (MDPI format, after manuscript body): "This research r eceived no external funding." Reported per MDPI/JCM journal requirem ents.	**YES**

Reference: von Elm E, Altman DG, Egger M, Pocock SJ, Gøtzsche PC, Vandenbroucke JP; STROBE Initiative. The Strengthening the Reporting of Observational Studies in Epidemiology (STROBE) statement: guidelines for reporting observational studies. PLoS Med. 2007;4(10):e296.

Checklist available at: https://www.equator-network.org/reporting-guidelines/strobe/
